# Thoracic magnetic resonance imaging in non-tuberculous mycobacterial pulmonary disease: Characteristics and potential implementation

**DOI:** 10.1371/journal.pone.0325347

**Published:** 2025-06-23

**Authors:** Ieuan Edward Shepherd Evans, Timothy Baird, Charles S. Haworth, Christopher Johnson, Helen Barker, Uta Hill, Dorothy Grogono, Odiri Eneje, Nicholas Screaton, Andres Floto

**Affiliations:** 1 Cambridge Centre for Lung Infection (CCLI), Royal Papworth Hospital, Cambridge, United Kingdom; 2 Thoracic Medicine Department, The Prince Charles Hospital, Queensland, Australia; 3 Faculty of Medicine, University of Queensland (UQ), Queensland, Australia; 4 Respiratory Department, Sunshine Coast University Hospital, Queensland, Australia; 5 Sunshine Coast Health Institute (SCHI), Queensland, Australia; 6 University of the Sunshine Coast (UniSC), Queensland, Australia; 7 Heart Lung Research Institute, University of Cambridge, Cambridge, United Kingdom; 8 Radiology Department, Royal Papworth Hospital, Cambridge, United Kingdom; 9 Molecular Immunity Unit, Department of Medicine, University of Cambridge, Cambridge, United Kingdom; The University of Georgia, UNITED STATES OF AMERICA

## Abstract

**Background:**

Non-tuberculous mycobacteria (NTM) are pulmonary pathogens with increasing incidence and prevalence worldwide, with people with cystic fibrosis (pwCF) traditionally considered at high risk of disease development. The imaging assessment of NTM-pulmonary disease (NTM-PD) relies heavily on high-resolution computed tomography (HRCT). However, due to lengthy treatment regimens and the need for long-term follow-up, serial HRCT’s result in progressive exposure to ionizing radiation; a particular concern in younger people.

**Methods:**

We performed a retrospective cohort study of patients who had undergone serial thoracic magnetic resonance imaging (tMRI) scans to monitor NTM-PD as a novel tool to image the lung with a view to creating an algorithm for the utility of tMRI in the management of NTM-PD.

**Results:**

Thirty-six patients, of which twenty-four had a diagnosis of CF, with suspected or confirmed NTM-PD underwent serial tMRI between 1^st^ January 2013 and 30^th^ June 2018. A total of 117 serial tMRI’s were performed (mean number per patient 3.25; range 2–6). The associated clinical impact that each serial MRI had on management, deemed as the utility of tMRI, found that all tMRI’s were classified as aiding management with 60 (51.3%) altering management. tMRI’s were more likely to alter management in the non-CF cohort than the CF cohort (69.4% vs. 43.2%). No imaging-related adverse events were reported across the 117 tMRI’s.

**Conclusion:**

This study highlights that tMRI may hold promise as a monitoring tool in NTM-PD and should be prospectively evaluated in the monitoring of individuals with NTM-PD.

## Introduction

Non-tuberculous mycobacteria (NTM) are environmental organisms that can cause difficult to treat lung infection in susceptible individuals. Rates of pulmonary NTM infection have been increasing worldwide, with people with cystic fibrosis (pwCF) traditionally considered at high risk of disease [1–4]. However, isolation of NTM species from microbiological samples does not necessarily always progress to inflammatory lung disease, referred to as NTM-pulmonary disease (NTM-PD). Therefore, deciding who requires treatment remains challenging and relies on the presence of positive microbiology (at least a single positive bronchoscopic sample or two positive sputum samples), clinical symptoms attributable to NTM infection and consistent radiological features [5,6]. These radiological features include bronchiectasis, nodules, tree-in-bud changes, consolidation, and cavitation [7–10]. High-resolution CT (HRCT) remains the gold standard imaging modality and has been shown to be more sensitive than chest x-ray for the detection of bronchiectasis, nodular infiltrates and cavities [5–7,11]. However, serial tracking of radiological changes using CT scanning before treatment initiation, during long term multi-drug treatment regimens, and during post-treatment follow-up is limited by cumulative exposures to ionising radiation. While the introduction of cystic fibrosis transmembrane regulator (CFTR) modulator therapies had been associated with improved disease outcomes and reduced risk of acquisition of NTM [12–14], the development of NTM-PD remains of concern and the challenge of obtaining longitudinal sputum samples for disease surveillance in pwCF on modulators has increased the importance of tracking radiological changes as a marker of NTM disease activity [15–17]. Thereby motivating the need for consideration of alternative imaging modalities to CT for NTM-PD.

Thoracic magnetic resonance imaging (tMRI), a radiation free modality, is emerging as a novel tool to image the lungs with recent studies confirming its non-inferiority to HRCT when assessing certain pulmonary diseases [12,13,18–24]. However, to date the evaluation of tMRI in assessing NTM-PD in individuals remains limited. Whilst MRI has been explored as a modality in pwCF to quantify lung disease severity, the evaluation of tMRI as an imaging modality for NTM-PD remains limited to small studies of *Mycobacterium avium* complex (MAC) infection in non-CF populations [5,13,25–27].

These new challenges in monitoring disease trajectories and treatment responses in pwCF, together with the likelihood for an increased reliance on cross-sectional imaging in disease surveillance, motivated us to further examine the potential role of tMRI in the management of patient at risk of NTM infection. Therefore, from the 1^st^ January 2013 onwards, the standard of care at the Royal Papworth Hospital for suspected or confirmed NTM-PD, has been to undergo an initial paired HRCT/tMRI (to first determine if tMRI is effective for detecting imaging changes of NTM infection at an individual level), followed by serial tMRI surveillance where appropriate.

This study describes our implementation of tMRI in the assessment of NTM-PD, outlines the tMRI features of NTM infection, and proposes an algorithm that incorporates tMRI in the longitudinal evaluation of all individuals, including pwCF, with NTM-PD.

## Methods

We performed a retrospective sequential cohort study of patients who had undergone serial tMRI scans to monitor NTM-PD at the Cambridge Centre for Lung Infection (CCLI), Royal Papworth Hospital, Cambridge, UK between 1^st^ January 2013 and 30^th^ June 2018. Clinical data was accessed and obtained between 1^st^ September and 1^st^ December 2018.

Paired tMRI and CT imaging at baseline formed part of usual ‘standard of care’ at the CCLI during the study period. All patients under the care of the CCLI service who had undergone a paired (or serial) tMRI and CT for NTM-PD during this period were identied. Imaging modalities were reviewed weekly at a multidisciplinary team meeting including expert thoracic radiologists, CF/bronchiectasis and microbiology physicians. Medical and laboratory records were reviewed to obtain clinical data including microbiology results (NTM species; smear and culture status; non-NTM cultures); spirometry (FEV1; FEV1% predicted); symptoms; current NTM treatment; and whether a diagnosis of CF had been made.

During the study period, a Siemens Magnetom Avanto 1.5 T MRI machine was used for tMRI imaging, using a protocol described in **Table 1**. tMRI findings were classified into the following categories: bronchiectasis; cavities; nodules; consolidation; atelectasis; pleural changes; bronchial wall thickening, mucous impaction; and lymphadenopathy. Additionally, each tMRI was recorded as being consistent or not with the 2007 ATS/IDSA HRCT criteria for the diagnosis of NTM-PD [6].

**Table 1:** Thoracic MRI protocol (Siemens Magnetom Avanto 1.5 MRI Scanner) at Royal Papworth Hospital, with diagnostic utility.

Sequence^*^	Specials	Plane	TR (ms)	TE (ms)	ST (mm)	DF (%)	Slices (mm^2^)	FOV (mm^2^)	Matrix	Voxel (mm)	Scan time (min:s)	Diagnostic Utility
T2 FSE (HASTE)	BH	cor	600	31	8	0	26	400 x 400	256 x 256	1.6 x 1.6	0:16	Infiltrates
T2 FSE (HASTE)	BH	tra	600	31	8	0	35	400 x 400	256 x 256	1.6 x 1.6	0:21	Infiltrates
T2 BLADE	mBH; FS	tra	3050	116	8	25	26	400 x 400	256 (85.7%)	1.6 x 1.6	1:04	Nodules; masses; lymph nodes; bones
T2 BLADE	mBH; FS	cor	3050	121	8	25	22	400 x 400	320 (75%)	1.3 x 1.3	1:04	Nodules; masses; lymph nodes; bones
T1/2 bSSFP		cor	437	1.16	4	−50	100	400 x 400	256 x 256	1.6 x 1.6	0:44	Ventilation; embolism
T1 GE (VIBE)	BH	tra	3.30	1.18	4	20	72	400 x 350	256 x 256	1.6 x 1.6	0:21	Nodules; masses; airways
T1 GE (VIBE)	BH; FS	tra	3.30	1.18	4	20	72	400 x 350	256 x 256	1.6 x 1.6	0:24	Nodules; masses; airways
T1 GE (VIBE)	BH	cor	3.30	1.18	4	20	52	400 x 350	256 x 256	1.6 x 1.6	0:18	Nodules; masses; airways
T1 GE (VIBE)	BH; FS	cor	3.30	1.18	4	20	52	400 x 350	256 x 256	1.6 x 1.6	0:22	Nodules; masses; airways
Perfusion (TWIST)	40 measurement; I.V. contrast	cor	1.72	0.70	5	(3d)	40	500 x 500	256 x 138	3.6 x 2.0	1:07	Perfusion
T1 GE (VIBE) ce	BH; FS	tra	3.30	1.18	4	20	72	400 x 350	256 x 256	1.6 x 1.6	0:24	Nodules; masses; airways
T1 GE (VIBE) ce	BH; FS	tra	3.30	1.18	4	20	52	400 x 350	256 x 256	1.6 x 1.6	0:22	Nodules; masses; airways

*BH – Breath hold; BLADE – Siemens rotating phase encoding sequence technique; bSSFB – balanced steady-state free precession; ce – Contrast enhanced; cor – Coronal; FS – Fat saturation; FSE – Fast spin echo; HASTE – single-shot half-Fourier fast spin-echo technique; GE – Gradient echo; mBH – Modified breath hold; Min – minutes; S – seconds; T1 – T1 weighted; T2 - T2 weighted; tra – Transverse; TWIST – fast gradient echo technique with view sharing; VIBE – Volumetric integrated gradient echo technique.*

* All sequences employed parallel imaging (iPAT) acceleration factor = 2.

Each tMRI was further classified as either a baseline scan (i.e., the initial paired HRCT/tMRI) or one showing disease stability, improvement, deterioration, or a mixed outcome. The utility of each tMRI in assessing NTM-PD was additionally evaluated by whether it ‘aided management’ (where the tMRI, in combination with clinical and microbiological parameters, aided future decisions about management, but did not change decision making independently) or ‘altered management’ (where the tMRI result by itself changed management, altered decision making about future treatment, prompted further investigations, or guided bronchoscopic sampling).

Ethical approval for the study was obtained through the Royal Papworth Hospital NHS Foundation Trust, project reference: S02444. The study was deemed to be a retrospective evaluation of low risk with no material ethical issues. The requirement for consent was waived by the ethics committee.

## Results

Thirty-six patients, of which twenty-four had a diagnosis of CF, with suspected or confirmed NTM-PD underwent serial tMRI between 1^st^ January 2013 and 30^th^ June 2018. A total of 117 serial tMRI’s were performed (mean number per patient 3.25; range 2–6).

### Clinical characteristics

Of the 117 tMRI scans, 65 (55.6%) were performed in patients with *M. abscessus* complex (MABS); 24 (20.5%) in patients co-infected with *M. avium* complex *(*MAC) and MABS; 23 (19.6%) in those infected with MAC alone; and 5 (4.3%) in patients with *M. kansasii*. Sixty-nine (59%) of patients were classed as being symptomatic at the time of MRI (CF 56.8%; non-CF 63.9%) with 57 (48.7%) being on NTM targeted treatment (CF 56.8%; non-CF 30.6%). A summary of the patients clinical and microbiological characteristics are shown in **Table 2**.

**Table 2:** Corresponding clinical and microbiological characteristics of 117 serial thoracic MRI’s performed in thirty-six patients to monitor NTM-PD.

Clinical Characteristic	Total tMRI n = 117 (%)	CF tMRI n = 81 (%)	Non-CF tMRI n = 36 (%)
**NTM Species**			
MAC	23 (19.7)	5 (6.2)	18 (50.0)
MABS	65 (55.6)	54 (66.7)	11 (30.6)
MAC/ MABS	24 (20.5)	22 (27.2)	2 (5.6)
*M. kansasii*	5 (4.3)	0 (0)	5 (13.9)
**Microbiology**			
Smear Positive	23 (19.7)	19 (23.5)	4 (11.1)
Culture Positive	80 (68.3)	55 (66.9)	25 (69.4)
**Other Pathogens**			
*P. aeruginosa*	51 (43.5)	45 (55.5)	6 (16.6)
*MSSA/MRSA*	15 (12.8)	15 (18.5)	0 (0)
*Aspergillus sp.*	12 (10.3)	11 (13.5)	1 (2.7)
*Serratia marcescens*	8 (6.8)	7 (8.6)	1 (2.7)
*S. maltophilia*	6 (4.1)	5 (6.2)	1 (2.7)
*Achromobacter sp.*	2 (1.7)	0 (0)	2 (5.6)
*H. influenzae*	4 (3.4)	1 (1.2)	3 (8.3)
*Moraxella catarrhalis*	1 (0.9)	0 (0)	1 (2.7)
*Streptococcus sp.*	4 (3.4)	0 (0)	4 (11.1)
*Escherichia coli*	1 (0.9)	0 (0)	1 (2.7)
*Enterobacter sp.*	1 (0.9)	0 (0)	1 (2.7)
*Klebsiella oxytoca*	1 (0.9)	0 (0)	1 (2.7)
*Tricosporon sp.*	2 (1.7)	2 (2.4)	0 (0)
***Lung Function***			
FEV1 (% predicted) ±SD	73.5 ± 4.0	71.8 ± 3.6	83.2 ± 18.1
***Symptomatic*** ^*^			
Yes	69 (59)	46 (56.8)	23 (63.9)
***On Treatment*** ^**^			
Yes	57 (48.7)	46 (56.8)	11 (30.6)

FEV1 – Forced expiratory volume in 1 second; MABS – *Mycobacterium abscessus* complex; MAC – *Mycobacterium avium* complex; *M. kansasii* – *Mycobacterium kansasii*; *P. aeruginosa* – *Pseudomonas aeruginosa*; MSSA – Methicillin susceptible *Staphylococcus aureus*; MRSA – Methicillin resistant; *sp*. – species; *Staphylococcus aureus*; *S. maltophilia* – *Stenotrophomonas maltophilia*; *H. influenzae* – *Haemophilus influenzae*; tMRI – thoracic magnetic resonance imaging; CF – Cystic fibrosis; non-CF – non-cystic fibrosis; NTM – non-tuberculous mycobacteria.

* *Symptoms consistent with possible NTM infection at the time of MRI – cough; sputum; weight loss; sweats, fatigue; malaise,* ** *On active NTM-PD treatment at the time of tMRI.*

### Thoracic MRI characteristics

One-hundred of the 117 tMRI’s (85.5%) met the 2007 ATS/IDSA HRCT criteria for NTM-PD (CF 88.9%; non-CF 77.8%) [6]. All patients had radiologically-defined bronchiectasis. Nodules were seen in 101 scans (86.3%); cavitation in 23 (19.7%); consolidation in 46 (39.3%); mucous impaction in 107 (91.5%); and bronchial wall thickening in 81 (69.2%).

**Fig 1:**
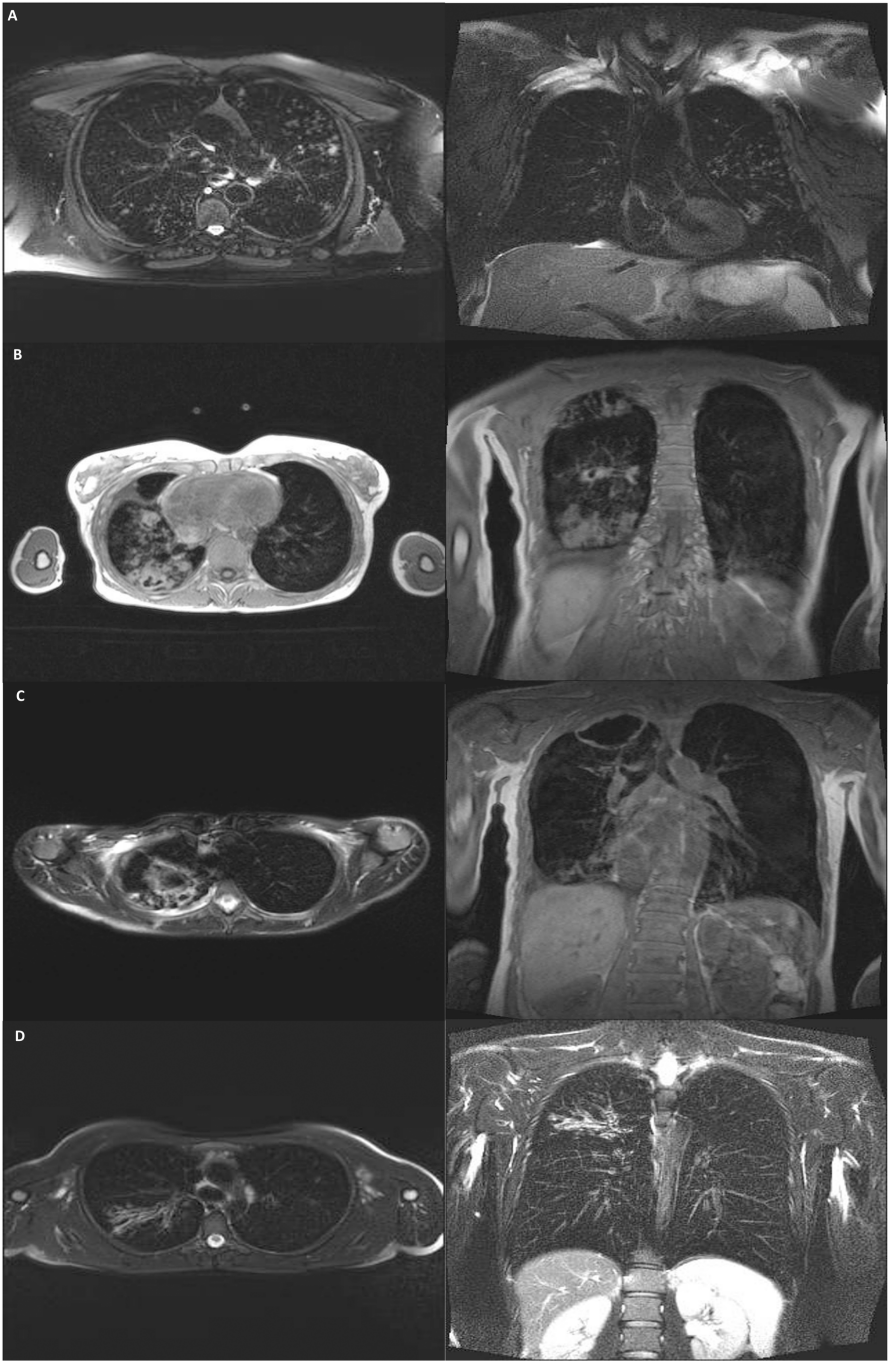
A: Thoracic MRI (T2 BLADE; transverse and coronal views) demonstrating bronchiectasis with branching nodular infiltrates in the left upper and right lower lobes, B: Thoracic MRI (VIBE; transverse and coronal views) demonstrating bronchiectasis with right upper and lower lobe cavities, nodular infiltrates, consolidation and pleural thickening, C: Thoracic MRI (VIBE; transverse and coronal views) demonstrating bronchiectasis with right upper lobe cavities with surrounding nodular infiltrates, D: Thoracic MRI (T2 BLADE; transverse and coronal views) demonstrating bronchiectasis with marked right upper lobe bronchial wall thickening and distal mucoid impaction.

Evaluating serial tMRI’s over time, we found that 28 (23.9%) demonstrated improvement; 19 (16.2%) remained stable; 24 (20.5%) showed evidence of progressive disease; and 10 (8.5%) revealed mixed changes, with some areas of improvement and some of progression. The associated clinical impact that each serial MRI had on management, deemed as the utility of tMRI, found that all tMRI’s were classified as ‘aiding management’ with 60 (51.3%) ‘altering management.’ tMRI’s were more likely to alter management in the non-CF cohort than the CF cohort (69.4% vs. 43.2%). No imaging related adverse events were reported across the 117 tMRI’s. Examples of the radiological features highlighted by tMRI are shown in **Fig 1**.

A summary of the tMRI radiological characteristics is outlined in **Table 3**.

**Table 3:** Radiological features of 117 serial thoracic MRI’s performed in 36 patients to monitor non-tuberculous mycobacterial pulmonary disease.

Radiological Characteristic	Total tMRI n = 117 (%)	CF tMRI n = 81 (%)	Non-CF tMRI n = 36 (%)
**MRI Findings**			
ATS/IDSA Criteria	100 (85.5)	72 (88.9)	28 (77.8)
Bronchiectasis	117 (100)	81 (100)	36 (100)
Nodules	101 (86.3)	72 (88.9)	29 (80.6)
Cavities	23 (19.7)	15 (18.5)	8 (22.2)
Consolidation	46 (39.3)	29 (35.8)	17 (47.2)
Pleural Changes	15 (12.8)	3 (3.7)	12 (33.3)
Bronchial Thickening	81 (69.2)	72 (88.9)	9 (25)
Mucous Impaction	107 (91.5)	80 (98.8)	27 (75)
Lymphadenopathy	37 (31.6)	29 (35.8)	8 (22.2)
**MRI Serial Comparison**			
Baseline	36 (30.8)	24 (29.6)	12 (33.3)
Improved	28 (23.9)	23 (28.4)	5 (13.9)
Stable	19 (16.2)	12 (14.8)	7 (19.4)
Progressed	24 (20.5)	14 (17.3)	10 (27.8)
Mixed	10 (8.5)	8 (9.9)	2 (5.6)
**MRI Utility**			
Aided Management^*^	117 (100)	81 (100)	36 (100)
Altered Management^**^	60 (51.3)	35 (43.2)	25 (69.4)

tMRI – thoracic magnetic resonance imaging; CF – Cystic fibrosis; non-CF – non-cystic fibrosis; ATS/IDSA criteria^6^

* *Defined as the tMRI, in combination with clinical and microbiological parameters, aided future decisions about management, but did not change decision-making on its own.*

** *Defined as the tMRI independently changed management or altered decision-making about future treatment or the need for further investigations. This was inclusive of the tMRI being utilized to guide targeted broncho-alveolar lavage for clarification of culture conversion vs. ongoing infection when required.*

## Discussion

To our knowledge, this is the largest series of tMRI’s analysed for the specific assessment of NTM infection in individuals with bronchiectasis or CF. Of the 117 serial tMRI’s obtained from thirty-six adults with suspected or confirmed NTM-PD, twenty-four had a confirmed diagnosis of CF. Therefore, this study is the first to describe the use of tMRI to perform serial monitoring of NTM disease caused by MABS, MAC and *M. kansasii*, with only one prior study comparing CT vs. MRI to assess culture confirmed MAC pneumonia in non-CF individuals [13]. The high proportion of MABS isolates is likely explained by the proportion of pwCF, in part related to the previously reported MABS outbreak within our CF centre [27,28].

At the time of MRI imaging, 59% of subjects reported symptoms consistent with possible NTM-PD and 48.7% of all subjects were undergoing NTM treatment. The proportion of symptomatic individuals is not unexpected as it is recognised that not all individuals with positive NTM isolates or radiologically defined disease have symptoms, and not all require NTM treatment [5]. Co-infection with other microorganisms was as expected given the underlying diagnoses of CF and bronchiectasis, with *Pseudomonas aeruginosa*, *Staphylococcus aureus* and *Aspergillus fumigatus* predominating. The frequency of coinfection was similar across both our CF and non-CF cohorts. Despite the recognition of concomitant infection with NTM and fungi, namely *Aspergillus* [29], there is a general paucity of literature reporting on the incidence of concomitant pathogens in NTM-PD. However, it is none-the-less important to note as they undoubtably have potential implications when interpreting the radiological changes outlined in our study.

### Radiological characteristics

The specific lung MRI protocol used at our centre is outlined in **Table 1**. Similar MRI protocols have been used in previous clinical trials and clinical practice for the short and long term monitoring of CF lung disease, in addition to a recently published trial in non-CF individuals with MAC pneumonia [13,30–33]. Using this protocol, MRI has been shown to successfully visualize bronchiectasis, bronchial wall thickening, mucous impaction, air fluid levels, consolidation and nodules, changes commonly seen across both our CF and non-CF cohorts (**Table 3**) [30,33].

### Bronchiectasis

Although NTM-PD is known to occur in individuals without bronchiectasis, particularly in the fibrocavitary form of disease, all subjects in our study were defined radiologically as having bronchiectasis on tMRI. Bronchiectasis is an established cause and/or result of NTM infection and a consistently reported HRCT finding in NTM-PD [5]. This may be explained by our cohort largely consisting of pwCF, in addition to our centre being a specialist referral base for individuals with bronchiectasis.

### Nodular infiltrates

Previously published data comparing the sensitivity and specificity of tMRI with CT has shown that MRI achieves both high sensitivity and specificity for various different sizes of pulmonary nodules, as small as 5 mm [5], and has therefore been determined to be an effective alternative to CT for diagnostic purposes in certain conditions, namely CF [34–36]. Despite this, concerns remain about its ability to detect smaller nodular infiltrates commonly seen in NTM infection [5]. However, it has been demonstrated that there is good agreement between HRCT and MRI in the detection of nodules in patients with MAC pneumonia [13]. Certainly, in our cohort, nodules were detected in more than 80% of tMRI’s across both the CF and non-CF cohort, supporting the feasibility of detecting smaller nodules associated with NTM infection.

### Cavities & consolidation

The high soft tissue contrast of tMRI allows for improved differentiation of fluid or inflammation in airways, alveoli and cavities compared with HRCT [13,20]. Both cavities and pulmonary consolidation are easily visualized with tMRI due to their high T2-weighted signal and have shown to be at least comparable to HRCT [5]. In our study, approximately 40% and 20% had evidence of consolidation and cavities respectively, higher than that reported in earlier studies [13].

### Bronchial wall thickening & mucous impaction

Importantly, tMRI may be superior to HRCT for determining bronchial wall thickening with a high signal on T2 weighted images representing oedema, and enhancement on post contrast, fat-suppressed T1 weighted images representing inflammation, phenomena that cannot be easily depicted with HRCT [27,30]. This is supported by the high rates of bronchial wall thickening seen in our study. It will be important to determine if the same changes are as easily depicted by HRCT, a study directly comparing HRCT and MRI images in NTM infection is therefore required to answer this question.

Mucous impaction is also well visualized down to the smallest airways. It is recognized as a high T2 signal filling of the bronchus along its course with branching in the periphery, providing a grape-like or ‘tree in bud’ appearance [30,32,33]. Again, these findings were both depicted in our study, with particularly high rates of mucous impaction in the CF cohort, a not unexpected finding.

### Pleural changes & lymphadenopathy

Due to the excellent soft tissue contrast portrayed with MRI, assessment of pleural changes and mediastinal lymph nodes is potentially advantageous compared with HRCT [20]. Unique to our study, we have reported on the tMRI findings of pleural changes and lymphadenopathy. Although the presence of lymphadenopathy was similar across the CF and non-CF cohorts, a higher proportion of the non-CF cohort demonstrated pleural changes (33.3% vs. 3.7%). Although it is difficult to speculate on the reason behind this, it is an important finding as it may suggest that pleural reaction on tMRI may provide radiological support for active NTM infection in non-CF individuals.

### Utility of serial tMRI in clinical practice

**Fig 2:**
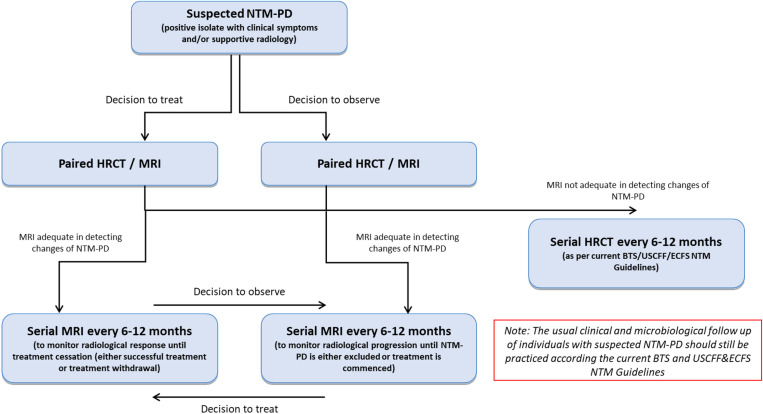
Proposed algorithm for the use of thoracic MRI (tMRI) to aid in the assessment and monitoring of individuals with suspected or confirmed non-tuberculous mycobacterial-pulmonary disease (NTM-PD). It is noted that not all individuals will be suitable for tMRI due to claustrophobia, implants and/or an inability to breath hold.

The utility of tMRI is highlighted by our study by both aiding with clinical decision making, whilst also providing the benefit of no radiation burden. Consequently, it could prove a useful additional tool to help evaluate people with NTM and help in (i) decisions to treat/not to treat and (ii) safely monitor treatment efficacy. Using this framework, we have developed an algorithm for the introduction of tMRI into the standard of care for individuals either under surveillance or being actively treated for NTM-PD (**Fig 2**). This algorithm is based on our own practice and experience in the assessment and monitoring of individuals with NTM-PD.

When reviewing individual serial tMRI’s of each subject, it was shown that 23.9% had improvement on radiology; 16.2% stability; 20.5% progression; and 8.5% mixed changes (**Table 3**). These results demonstrate that radiological changes of NTM-PD over time can be adequately depicted using tMRI. More importantly, all 117 tMRI’s were deemed by our authors to ‘aid management’ with 51.3% ‘altering management’ (69.4% in the non-CF cohort) (**Table 3**). Although these definitions have been arbitrarily defined for this study, and potentially subject to bias, it still highlights the utility of tMRI in helping guide NTM-PD treatment at our centre.

Although there are currently no formal guidelines that dictate the frequency of imaging required in the monitoring of NTM-PD, is has been suggested that serial imaging with HRCT at least every 6–12 months is a reasonable approach [4,5,12]. We have adopted this practice at our centre for individuals with suspected or confirmed NTM-PD, with them undergoing an initial paired HRCT and tMRI at the time of diagnosis. From this, if it is determined that tMRI is efficacious in determining the presence of NTM infection (i.e., comparable to the paired HRCT), then further monitoring should be performed with serial tMRI alone. The length of time between MRI’s should be 6–12 months as determined by the treating physician, and should be dependent on clinical progress, culture status, and whether the individual is under treatment or observation (**Fig 2**).

Finally, and perhaps of paramount importance, is the benefit of serial tMRI compared with HRCT in relation to ionizing radiation exposure. In our study, individuals underwent an average of 3.25 tMRIs with the maximum being six in four individuals, three of whom had CF. This number of serial scans is likely an underestimation and would certainly increase with the rising prevalence of NTM and the increasing requirements for lengthy treatment regimens. The potential reduction in cumulative radiation exposure and thus the reduction in long term malignancy risk, offered by tMRI compared to HRCT, makes tMRI an attractive alternative for monitoring individuals with chronic lung disease.

There are several limitations to our study. The retrospective nature of the study means that it is possible that not all individuals with NTM-PD were captured or underwent serial tMRI. Additionally, it is a single center study in adults only with all tMRI’s analysed by a radiologist with expertise in thoracic imaging and NTM infection. Although this could be seen as a strength, its findings may not be generalisable to other centres and/or patient cohorts. Moreover, the evaluation of tMRI as an imaging modality for NTM-PD remains significantly limited, particularly in CF [13]. Further studies directly comparing both imaging modalities are warranted to validate tMRI as a tool for monitoring NTM-PD. Finally, the cost and scanning time of tMRI compared to HRCT has not been discussed, though MRI scans are currently more expensive and take more time to perform and interpret. However, while this cost is reducing, the practicality of incorporating tMRI as routine practice when assessing NTM-PD at some institutions needs to be considered.

Despite these limitations, our current study demonstrates that tMRI can be safely and effectively used in the assessment of NTM-PD highlighting an exciting development in thoracic radiology and in the management of NTM infection.

## Conclusion

This study highlights the utility of tMRI as a radiation-free imaging modality that can be used in the assessment of NTM-PD. Our protocol outlines the way tMRI can be integrated into the standard of care for individuals likely to need prolonged disease surveillance. tMRI was able to visualise the hallmarks of NTM infection including bronchiectasis, nodules, tree-in-bud, consolidation, cavitation, mucous impaction, and bronchial wall thickening with direct impacts on treatment recommendations at our centre. While further benefits of tMRI include offering a safe means by which to monitor pwCF with NTM-PD who have experienced a reduced ability to readily provide sputum samples, as part of disease surveillance, since the introduction of CFTR modulator therapy. It provides the platform from which future prospective studies evaluating tMRI in NTM-PD could be implemented.

## Supporting information

S1 Data NTM_MRI Data_anonymised_IE. (XLSX)
